# Diversity of imaging features of ovarian sclerosing stromal tumors on MRI and PET-CT: a case report and literature review

**DOI:** 10.1186/s13048-018-0473-1

**Published:** 2018-12-20

**Authors:** Hiroki Matsutani, Go Nakai, Takashi Yamada, Kazuhiro Yamamoto, Masahide Ohmichi, Yoshifumi Narumi

**Affiliations:** 1 Department of Radiology, Osaka Medical College, 2-7 Daigaku-machi, Takatsuki, Osaka, 569-8686 Japan; 2 Department of Pathology, Osaka Medical College, 2-7 Daigaku-machi, Takatsuki, Osaka, 569-8686 Japan; 3 Department of Obstetrics and Gynecology, Osaka Medical College, 2-7 Daigaku-machi, Takatsuki, Osaka, Japan

**Keywords:** Sclerosing stromal tumor (SST), Magnetic resonance imaging (MRI), Positron emission tomography with computed tomography (PET-CT), Diffusion-weighted imaging (DWI)

## Abstract

**Background:**

Sclerosing stromal tumors (SST) are rare, benign tumors classified as sex cord stromal tumors. To our knowledge, positron emission tomography with computed tomography (PET-CT) findings of SST have only been described in one report and imaging findings on diffusion-weighted imaging (DWI) have only been described in three reports. Characteristic imaging features of SST on PET-CT and DWI have not yet been identified. Here we report a case of multilocular SST with solid components showing mild FDG uptake and slight hyperintensity on DWI, and reviewed the literature.

**Case presentation:**

Seventeen-year-old woman presented with a complaint of abdominal pain and was admitted due to infectious colitis. Ultrasonography incidentally revealed a multiseptated cystic mass in the pelvis. Magnetic resonance imaging (MRI) showed a large multilobulated cystic mass with irregularly thickened septa and solid components originating in the left adnexa. On T2WI, the cystic components had the same signal intensity (SI) as water, and the irregularly thickened septa and solid components showed intermediate SI higher than the SI of the uterine myometrium. The septa and solid components also showed early strong enhancement on contrast-enhanced T1WI and slight hyperintensity on DWI. The PET-CT showed mild FDG uptake in the solid components of the tumor (SUV: 2.11). According to previous articles, the morphology of SSTs are various; solid mass, well-circumscribed multilobular mass, well-demarcated mass, and multilocular cysticmass. According to the reports describing DWI findings of SST, the SI varies from significant hyperintensity to slightly hyperintensity like in this case. Only one report describing PET-CT findings of SST showed intense FDG uptake (SUV max: 7.0).

**Conclusion:**

The findings on DWI and PET-CT of our case and the past reports describing PET and DWI findings of SSTs are not consistent. The wide variety of the signal intensity on MRI and FDG uptake on PET could be due to the pathological diversity caused by the cellular areas undergoing collagenous sclerosis, which transforms the tumor into admixture of the collagen and the densely fibrous components with edema.

## Background

Sclerosing stromal tumors (SST) are benign ovarian neoplasms first described in 1973 by Chalvardjian and Scully [1]. Because SST have been observed in young adults, preoperative discrimination between SST and malignant tumors is important for fertility preservation. On MRI, they are known to be mainly heterogeneous solid tumors showing intermediate signal intensity (SI) with hyperintense foci corresponding to stromal edema on T2 weighted imaging (WI) and striking enhancement on contrast-enhanced T1WI [2].

To our knowledge, however, there has been only one report describing positron emission tomography with 2-deoxy-2-[fluorine-18]fluoro- D-glucose (FDG) integrated with computed tomography (PET-CT) findings of SST in the English-language literature [3] and three reports describing MR findings on diffusion-weighted imaging (DWI) [3–5]. Characteristic imaging features of SST on FDG-PET and MRI DWI have not yet been identified.

Therefore, here we report a case of SST with solid components showing mild FDG uptake (maximum standardized uptake value (SUV): 2.11) and slight hyperintensity on DWI.

## Case presentation

Seventeen-year-old woman, nulligravida, presented with a complaint of abdominal pain and was admitted due to infectious colitis. Ultrasonography incidentally revealed a multiseptated cystic mass in the pelvis. Family history and past medical history were unremarkable, and her menstrual cycle was regular. Blood cell counts and blood biochemistry were normal. Serum levels of alpha-fetoprotein (AFP), carcinoembryonic antigen (CEA) and carbohydrate antigen 19–9 (CA19–9) were all within normal limits, while cancer antigen 125 (CA-125) was elevated at 76.3 U/mL (normal range 0–35.0). Levels of serum hormones including estradiol (76.3 ng/ml; normal range, 22–144 ng/ml), luteinizing hormone (LH; 4.7mIU) /ml and follicle-stimulating hormone (FSH; 2.9 mIU/ml) were normal.

**Fig. 1:**
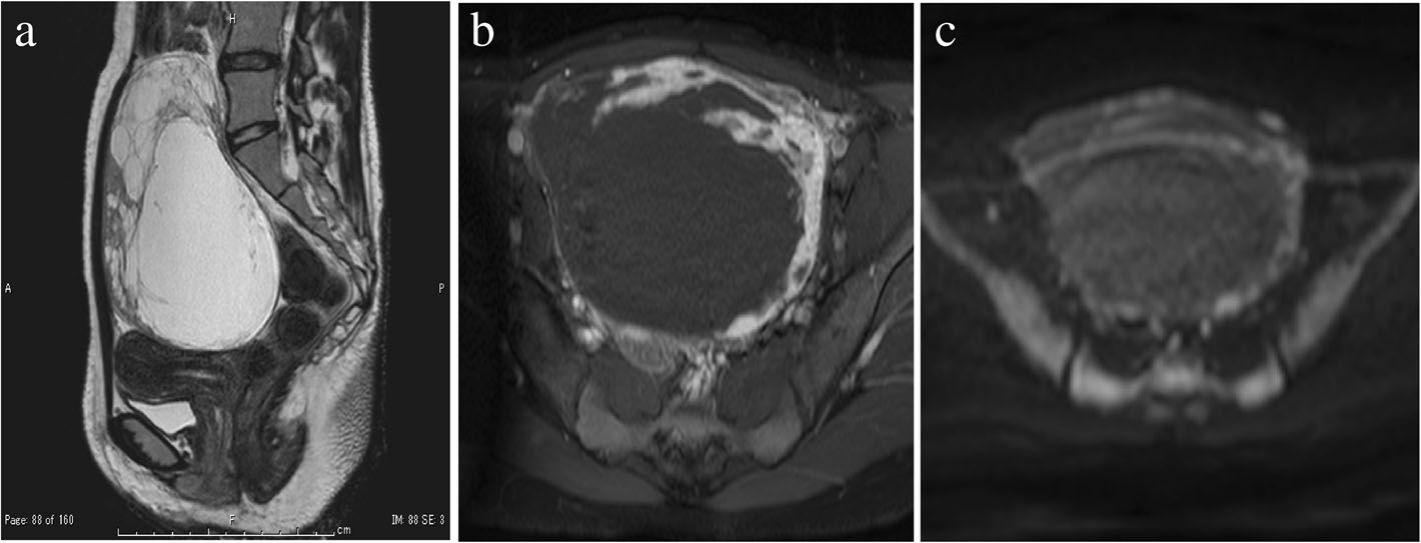
**a**. On sagittal T2-weighted imaging (repetition time [TR]/echo time [TE]: 1500/147.39 ms), the cystic components have the same signal intensity (SI) as water, and the irregularly thickened septa and solid components (arrows) have intermediate SI, higher than the SI of uterine myometrium. **b**. On axial contrast-enhanced T1-weighted imaging (repetition time [TR]/echo time [TE]: 534/13 ms), the septa and solid components (arrows) show early strong enhancement. **c**. On axial diffusion-weighted imaging (TR/TE: 1950/70 ms, b = 1000 s/mm2), the septa and solid components show slight hyperintensity

**Fig. 2:**
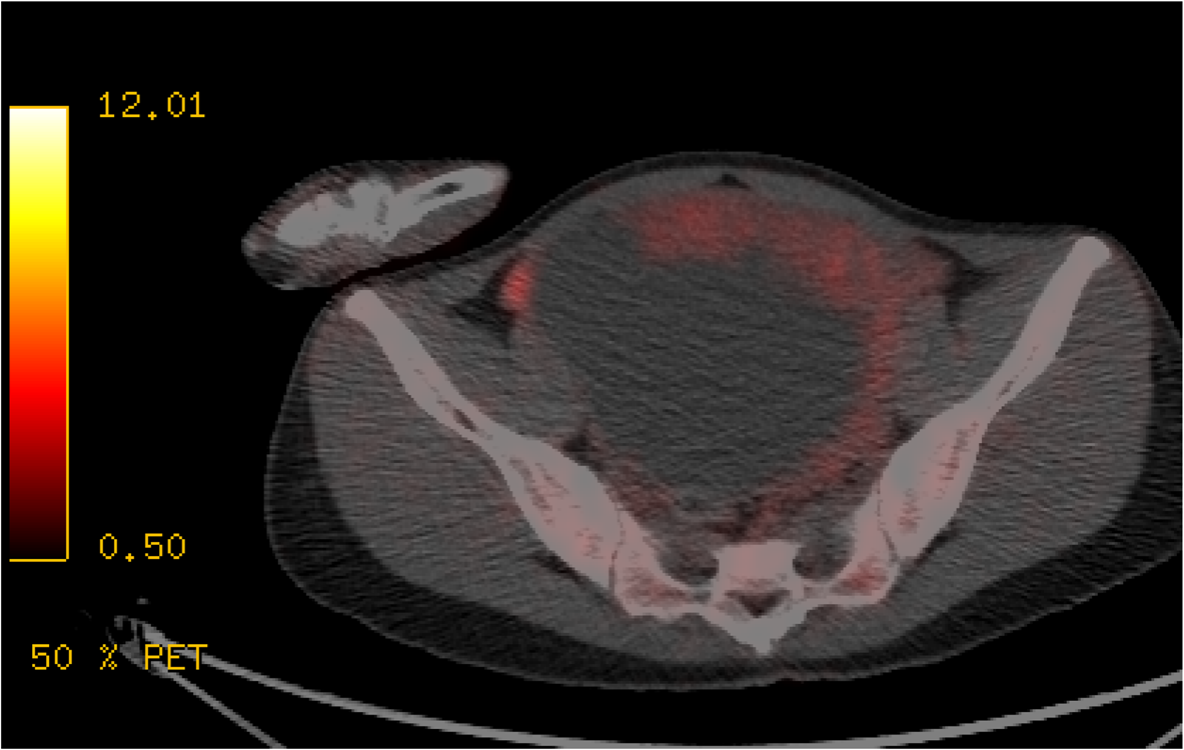
PET-CT shows mild FDG uptake in solid components of the tumor. (SUV: 2.11)

Pelvic magnetic resonance imaging (MRI) showed a 141 × 96 × 127-mm well-demarcated multilocular cystic mass with irregularly thickened septa and solid components originating in the left adnexa. On T2WI, the signal intensities of the cystic components had the same SI as water, and those of the irregularly thickened septa and solid components had intermediate SI, higher than the SI of uterine myometrium (Fig. 1a). On T1WI, the septa and solid components had slight higher SI than uterine myometrium and showed early strong enhancement on contrast-enhanced T1WI (Fig. 1b) and slight hyperintensity on DWI (Fig. 1c). PET-CT showed mild FDG uptake in solid components of the tumor (SUV: 2.11) (Fig. 2).

The preoperative imaging diagnosis was SST or granulosa cell tumor, and it was not likely to be associated with a malignant ovarian tumor because of its low FDG uptake.

The patient underwent left oophorectomy and omentectomy. The cystic mass originated in the left ovary and measured 15 cm in diameter. Grossly, some thickened septa were observed in the mass but there were no obvious solid components. The cysts contained clear, straw-colored fluid.

**Fig. 3:**
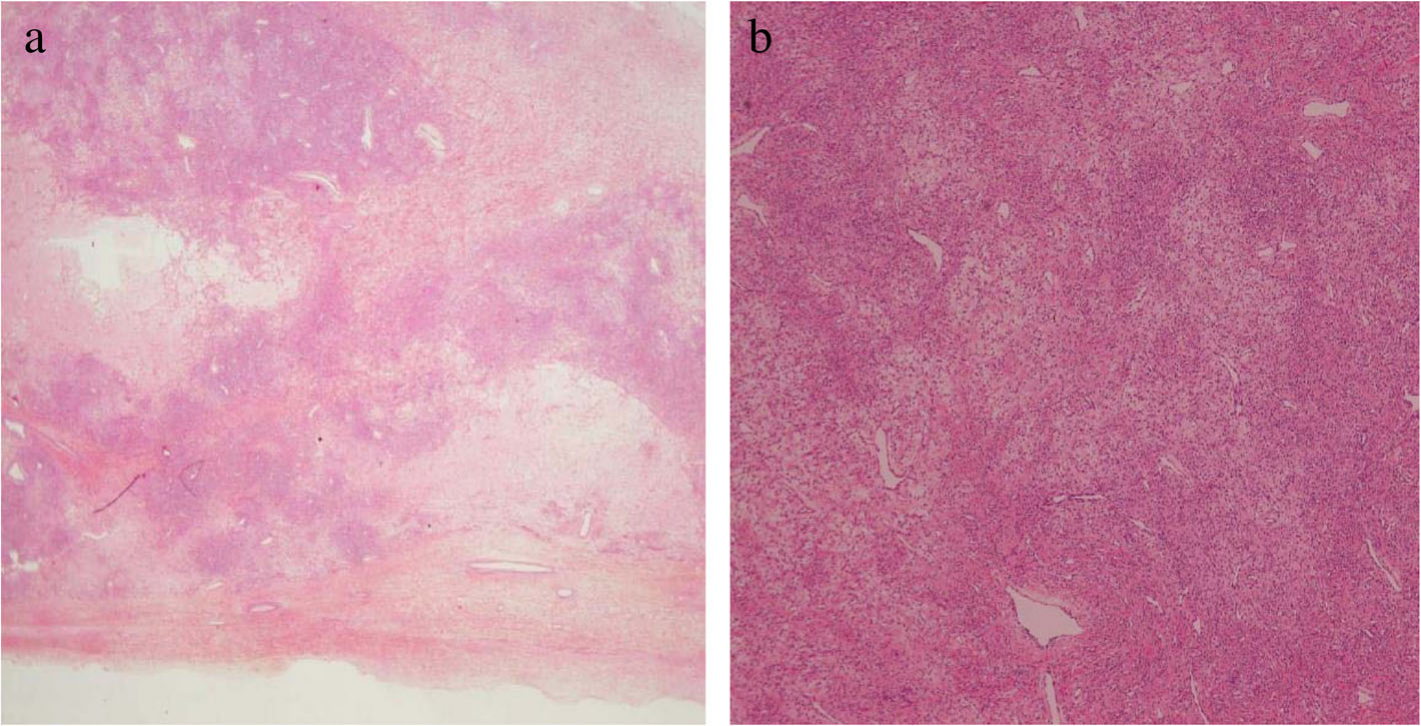
**a**. Low power microscopic examination shows admixture of cellular areas, densely fibrous and edematous tissue producing a pseudolobular appearance. **b**. Higher power image of cellular area. Cellular area mainly consists of rounded cells with an epithelial appearance and spindle-shaped cells. The cells vary in size and mix in a haphazard arrangement. Prominent vascular networks with a hemangiopericytomatous pattern are observed

Histological examination revealed that the mass consisted of hypercellular areas with prominent vascular networks separated by hypocellular areas that corresponded to collagenous and edematous areas, or a so-called “pseudolobular appearance”. Coarse collagenous fibers surrounding individual cells formed collagen bundles between cells, leading to heterogeneous cell density even in cellular areas (Fig. 3a). The hypercellular areas were composed of a dual cell population of collagen-producing bland spindled cells and rounded epithelioid cells. Prominent vascular networks with a hemangiopericytomatous pattern were observed (Fig. 3b). Immunohistochemically, tumor cells were positive for α–inhibin. Thus, the final histological diagnosis was SST of the ovary.

### Review of the literature

We searched the PubMed database for previous cases published in English from 1966 to September 2017 with the terms of “sclerosing stromal tumor”, “ovary” and “imaging”.

The results showed only one report describing PET findings for SST and three reports describing imaging findings on DWI for SST, including the above-mentioned report describing PET findings. Imaging findings on PET and DWI are summarized in Table 1.

**Table 1:** The summary of imaging findings for sclerosing stromal tumor on PET-CT and diffusion-weighted image(DWI) including previous reports

Author (year)	Morphology	DWI	PET-CT	Reference
Ito (2017)	solid mass	significant hyperintensity	none	4
Tomimatsu (2016)	well-circumscribed multilobular mass	hyperintensity	high FDG uptake	3
Wada (2009)	well-demarcated mass	slight hyperintensity	none	5
Our case	multilocular cysticmass	slight hyperintensity	mild FDG uptake	/

According to one report describing PET findings of SST, the tumor showed intense FDG uptake (SUV max: 7.0). Although SST tended to show high SI on DWI, the exact degree of high SI varied between cases.

## Discussion and conclusions

SST of the ovary are rare, benign tumors classified as sex cord stromal tumors. The tumor tends to affect younger women predominantly in their 20s and 30s, in contrast with other sex cord stromal tumors such as thecomas and fibromas, which are most common in women in their 50s and 60s [1]. Preoperative discrimination between SST and malignant tumors is crucial because SST tend to be solid tumors similar to malignant tumors and affect adolescents and young adults who require fertility preservation. Characteristic MRI findings of SST have made it feasible to differentiate them. According to previous articles, a pseudolobular structure comprising hypervascular cellular area is detected as hypointense nodules separated by hyperintense stroma corresponding to edematous and hypocellular areas on T2WI. In addition, SST show early marked enhancement on dynamic MRI, especially in the pseudolobulated area, reflecting their prominent vascularity. However, diversity in MR findings among SST has been observed as more cases of SST are being reported. The morphology of SST can be not only solid but also mainly cystic like in this case [6]. One case report of solid SST in a middle aged woman describes pseudolobulation showing slight hyperintensity separated by a hypointense hypocellular area on T2WI and gradual light enhancement on dynamic MRI [5]. The authors propose that the atypical imaging might be due to the presence of numerous collagen fibers in hypocellular areas and a large amount of thrombi in tightly packed cellular areas. In another case, the edematous or fibrous hypocellular area that was expected to show hyperintensity on T2WI was nearly isointense to the pseudolobules [4]. The authors of this report also proposed that the rich collagen fibers might contribute to the decreased signal of the background hypocellular area.

There have been three reports describing DWI of SST in which nodular solid areas in SST showed significant hyperintensity [4], hyperintensity [3] and slight hyperintensity [5] like in this case. Ito et al. suggest that DWI might be a promising technique for distinguishing pseudolobules with abundant tumor cells from edematous or collagenous background because hypercellular pseudolobules demonstrated significant hyperintensity compared to hypocellular background. However, the difference between hypercellular pseudolobules and hypocellular background can be obscure when pseudolobules show slight hyperintensity on DWI as in our case.

In this case, a malignant ovarian tumor was ruled out as a preoperative diagnosis due to the low FDG uptake. In contrast, only one report describing PET-CT findings of SST described that a solid component of the tumor that corresponded to well enhanced area on contrast-enhanced T1WI showed intense FDG uptake (SUV max: 7.0) [3]. Although they proposed that the PET positivity might be attributable to increased vascularity in SST, the thickened septa and solid components that were enhanced on contrast-enhanced T1WI did not demonstrate intense FDG uptake in this case.

The wide variety of morphology, signal intensity on MRI and FDG uptake on PET among SST might be affected by one of their characteristic features, that is, the tendency of the cellular areas to undergo collagenous sclerosis, producing a variegated appearance due to admixture of cellular, densely fibrous and edematous tissue on low power microscopic examination (Fig. 3a). Furthermore, coarse collagenous fibers that surround individual cells form collagen bundles between cells, leading to heterogeneous cell density even in cellular areas (Fig. 3b). We surmise that the inhomogeneous structure of cellular areas as well as haphazard arrangement of cellular areas, fibrous and edematous tissue might result in the high diversity of morphology, signal intensity on MRI and FDG uptake on PET among SST.

However, further studies on correlation between imaging findings on T2WI, DWI and FDG-PET and pathological examination are warranted because there have been only a few reports describing imaging features of SST on DWI and PET-CT.

## Abbreviations

AFP: Alpha-fetoprotein
CA-125: Cancer antigen 125
CA19–9: Carbohydrate antigen 19–9
CEA: Carcinoembryonic antigen
DWI: Diffusion-weighted imaging
FDG: 2-deoxy-2-[fluorine-18]fluoro- D-glucose
FSH: Follicle-stimulating hormone
LH: Luteinizing hormone
MRI: Magnetic resonance imaging
PET-CT: Positron emission tomography with computed tomography
SI: Signal intensity
SST: Sclerosing stromal tumor
WI: Weighted imaging
